# Advances in the Role of Sodium Hypochlorite Irrigant in Chemical Preparation of Root Canal Treatment

**DOI:** 10.1155/2023/8858283

**Published:** 2023-01-13

**Authors:** Chen Cai, Xuan Chen, Yang Li, Qianzhou Jiang

**Affiliations:** Department of Endodontics, Affiliated Stomatology Hospital of Guangzhou Medical University, Guangdong Engineering Research Center of Oral Restoration and Reconstruction, Guangzhou Key Laboratory of Basic and Applied Research of Oral Regenerative Medicine, Guangzhou, 510182 Guangdong, China

## Abstract

Irrigation of root canal system is of great significance to the success of endodontic treatment, where sodium hypochlorite (NaOCl) is the most widely used irrigant in chemical preparation. NaOCl functions by eliminating bacterial biofilms and dissolving organic tissue, which may vary according to several factors such as the microbiology of root canal infection and the concentration of the irrigant. It has been proposed that the effectiveness of NaOCl could be enhanced via several methods, including heating the irrigant, applying in conjunction with certain reagents, or activating by agitation techniques. Despite its antibacterial and tissue-dissolving capacities, NaOCl should be used with caution to avoid detrimental effect due to its cytotoxicity and negative effect on dentin properties. In this narrative review, we discussed the factors that affect the properties of NaOCl, the methods to improve its efficacy, and the side effects that might occur in clinical practice.

## 1. Introduction

The elimination of microorganisms, necrotic tissues, and accumulated hard tissue debris is pivotal to the success of endodontic treatment [1]. Since more than 35% of the root canal surface remained uninstrumented regardless of the instrumentation technique applied, chemical preparation using disinfecting solutions to irrigate the root canal system is considered necessary [2].

Sodium hypochlorite (NaOCl) is the most commonly used irrigant in endodontics [3]. It is regarded as the gold standard of root canal irrigants due to its antibacterial and tissue-dissolving capabilities [3]. Though the role of NaOCl in chemical preparation has been long established, it is still of great significance to investigate the factors that affect the effectiveness of NaOCl and methods of improvement, while minimizing the potential side effects. Over the last years, these aspects of the application of NaOCl have been constantly studied with emerging experimental techniques; the role of NaOCl in chemical preparation should be advancing with the times.

In an attempt to provide guideline for clinical practice and future studies, this paper is aimed at providing an overview of the published work discussing the parameters affecting the efficacy of NaOCl, clinical strategies that could enhance its effectiveness and side effects caused by NaOCl that could possibly occur during chemical preparation.

## 2. Microbiology of Root Canal Infections against NaOCl

Verification has been established regarding the etiologic role of intracanal microorganisms in the evolution of pulpal and periapical diseases, the elimination of which is a crucial function of NaOCl irrigant. The features of bacterial biofilm, including the bacterial species, biofilm structure, and maturity, could significantly influence the antibacterial effectiveness of NaOCl irrigant.

Numerous studies have investigated the NaOCl-resistant capability of different bacterial species *in vitro*. *Enterococcus faecalis* (*E. faecalis*) is one of the main pathogens of persistent endodontic infection and has been long used to test the efficiency of root canal irrigants [4]. Some bacterial species that also contribute to root canal infection were found to be more sensitive to NaOCl than *E. faecalis*. Darrag [5] revealed that *Streptococcus mutans* (*S. mutans*) was significantly more sensitive to 5.25% NaOCl compared to *E. faecalis* in planktonic condition. Using 0.5% NaOCl for 10 s lowered the colony-forming unit (CFU) below the limit of detection in the case of *Actinomyces naeslundii* (*A. naeslundii*) and *Candida albicans* (*C. albicans*), while it took 30 min for the same irrigant to reduce the CFU of *E. faecalis* to zero, suggesting that *E. faecalis* was more resistant to NaOCl [6]. In contrast, some other bacterial species was found to be equally sensitive to NaOCl as *E. faecalis.* Ghivari et al. [7] reported that *E. faecalis* and *Staphylococcus aureus* (*S. aureus)* were both 100% eliminated after 10 s of contact with 5.25% NaOCl *in vitro*. Moreover, the NaOCl-resistant capability of *E. faecalis* is also strain-related as Yang et al. [8] reported that the *E. faecalis* VP3-181 biofilms were more sensitive than Gel 31 biofilms when treated with 2% NaOCl.

The structure of bacterial biofilm plays an important role in its susceptibility against NaOCl irrigant, possibly due to the evolution of the microenvironment within the biofilm [9]. Matrix of the biofilm also plays an important role in the protection of biofilm bacteria against chemical and mechanical stresses and consequently a crucial role in *E. faecalis* survival [10]. Bacteria grown in a multispecies biofilm was found to be more resistant compared to that in a monospecies biofilm. Swimberghe et al. [11] compared the NaOCl susceptibility of a monospecies biofilm of *E. faecalis* with a multispecies biofilm containing *E. faecalis*, *Fusobacterium nucleatum* (*F. nucleatum*), *Prevotella intermedia* (*P. intermedia*), and *Porphyromonas gingivalis* (*P. gingivalis*) and found that *E. faecalis* was significantly less susceptible to NaOCl treatment in the multispecies biofilm, regardless of the NaOCl concentration applied (0.025%-2.5%). In addition, it has been reported that the resistance to NaOCl in a dual-species biofilm containing *S. mutans* was 30-fold higher than that of the *E. faecalis* single-species biofilm [12]. However, Yap et al. [13] revealed that no significant difference was found between the single- and multiple *E. faecalis* biofilm groups after 0.9% NaOCl was applied, suggesting that when *E. faecalis* is grown as part of a multispecies biofilm, it did not gain any further resistance to NaOCl.

The biofilm architecture, especially its bacterial compactness, is another influential factor that affects the antibacterial efficacy of NaOCl. 2% NaOCl had a significantly weaker effect on the bacterial dense biofilms with lower water and EPS content, resulting in even stiffer biofilms, as evidenced by the significant reduction of the biofilm stress relaxation, compared to biofilms which are richer in water and EPS [14]. An ensuing experiment revealed that treatment of the structurally compacted biofilms with NaOCl had a significant impact on the bacterial cell biofilm component without significantly affecting the EPS biofilm component, whereas treatment of the less compacted biofilm showed significant changes regarding both bacterial cell and EPS biofilm component [15].

The maturity of biofilm also contributes to its susceptibility to NaOCl irrigant. It has been widely reported that the relatively mature biofilms (formed for several weeks) were more resistant to NaOCl compared to young biofilms (formed for one day/several days) [8, 16, 17]. Notably, some researchers reported a difference between the NaOCl-resistant behavior of young and old biofilms. The antibacterial effect was concentration-dependent in terms of young biofilms, whereas a relatively high concentration was required to present a significantly greater antibacterial efficacy against mature biofilms [11, 18]. Wang et al. [19] found that 6% NaOCl dissolved significantly more 3-day-old biofilm than 3-week-old biofilm, whereas 2% NaOCl removed a similar percentage of young and old biofilms. However, Du et al. [20] found that significantly more cells were killed in young biofilms than in old biofilms when treated with both 2% and 6% NaOCl. The contrary results might be attributed to the difference in the maturity of the young biofilms (1 day vs. 3 days) in the relative studies.

Compared to younger biofilm, more viscous water molecules bounding to the EPS structure, along with more viscous EPS material, were found in mature biofilm, which could be a potential explanation of the age-dependent resistance to NaOCl [14]. The turning point of maturity, indicating by the time when biofilms become significantly more resistant to NaOCl irrigant, was reported to be about 2-3 weeks [18]. Sauer et al. [21] reported that it takes at least 9 days for monospecies biofilm to reach maturation.

## 3. Pulp Tissue Dissolution Capacity of NaOCl

The remnant pulp tissue could provide ideal conditions for microorganisms to survive and proliferate [22, 23]; the dissolution of pulpal remnant and organic tissue using NaOCl is of great significance to the success of root canal treatment [24].

All forms of chlorine in NaOCl solution, including hypochlorous acid (HOCl) and hypochlorite ion (OCl-), are collectively referred to as “free available chlorine”[25]. By direct contact with the organic matter, the free available chlorine molecules lead to amino acid degradation and hydrolysis, manifesting as tissue dissolution [26]. Moreover, unaltered NaOCl solution has a pH in the range of 11.5-12.5 depending on the concentration [25]; sodium hydroxide (NaOH) in such alkaline solution also contributes to the dissolution of organic tissue via saponification of fats [27]. NaOCl exerts a digestive effect on both vital and necrotic tissues [28, 29], while the dissolution effect on necrotic tissue was found to be greater than that on normal, healthy tissue [30].

It is noteworthy that EDTA reduces the tissue-dissolving capability of NaOCl due to the dramatic depletion of free available chlorine content [31, 32], as an almost complete loss of free available chlorine was observed immediately upon mixing NaOCl and EDTA [23]. In addition, the combination of EDTA and NaOCl would significantly decrease the NaOH level [33]. Despite the low level of NaOH in NaOCl solutions [34], this may partially explain the negative effect of EDTA on the tissue dissolution efficacy of NaOCl. Therefore, in order to perform its dissolution capacity on remnant pulp tissue, NaOCl should not be used with EDTA simultaneously.

Previous studies have reported that the presence of dentin has a detrimental effect on the ability of NaOCl to dissolve pulp tissue [35, 36]. This is related to the chemical composition of dentin which presents high concentrations of carbonate; the inorganic apatite and other dentin components are responsible for the buffering effect of dentin against acids and alkali [37]. A study of Cullen et al. [30] revealed that the presence of dentin did not have an impact on the tissue dissolution capability of 8.25% NaOCl if the irrigant was refreshed; the authors emphasized the importance of consistent refreshment of NaOCl irrigant to counteract the buffering effect of dentin.

Tissues from different sources were used to test the tissue dissolution effectiveness of NaOCl due to the availability, including bovine pulp and muscle as well as porcine pulp, muscle, and palatal mucosa [34, 38–42]. Experiments adopting human pulp tissue as study objects closely mimic the clinical conditions, though it is hard to unitize the samples [43], while the surface area of exposed tissue exerts an influence on the dissolution process [44]. The source of tissue is another influential factor, as it took 3 times longer for NaOCl to completely dissolve porcine palatal mucosa compared to human pulp [45]. This should be taken into account in the experimental design of *in vitro* studies.

The tissue-dissolving capability of NaOCl could also be influenced by various factors such as concentration, temperature, agitation, and pH, which will be discussed in the following sections.

In addition to the various uses mentioned above, NaOCl also possesses other functions in endodontic treatment. Tooth discoloration caused by the accumulation of hemoglobin or other forms of hematin molecules in dentinal tubules after endodontic treatment is a clinical esthetic problem [46]. Bleaching could be done using NaOCl to improve the appearance as NaOCl has a strong bleaching effect on blood-stained teeth [47]. Furthermore, NaOCl is advantageous in vital pulp treatment as it contributes to the removal of biofilm, cavity disinfection, and hemostasis [48]. A recent systematic review identified a clear trend towards the application of NaOCl for pulp wound lavage during vital pulp therapy in recent trials [49]. Studies by Taha et al. [50] and Simon et al. [51] have also highlighted the impact of wound surface disinfection by NaOCl on clinical outcomes of partial and full pulpotomies. Ballal et al. [52, 53] revealed that the application of NaOCl could reduce postoperative discomfort and painful early failure while improving the pulpal survival time after carious exposure and direct pulp capping.

## 4. Factors That Influence the Chemical Properties of NaOCl

### 4.1. Exposure Time

It is widely agreed that the increase of exposure time would lead to a significantly better antibacterial efficacy, though the testing exposure time varies from 1 min to 30 min due to the difference in experimental design.

Wang et al. [54] found that 3 min of exposure resulted in more dead bacteria in root canal than 1 minute of exposure to both 2% and 6% NaOCl. As for dentin disinfection, Ma et al. [55] reported that by increasing the exposure time from 1 min to 3 min, a significantly higher proportion of deal cell volume in dentin was achieved regardless of NaOCl concentration (1%, 2%, and 6%).

Petridis et al. [56] investigated the influence of exposure time on biofilm architecture and removal; the results demonstrated that 5 min of exposure time to 2% NaOCl led to significantly more biofilm disruption and dissolution compared to 2 min and 1 min. Nevertheless, another study showed that no difference in biofilm removal was seen between 5, 7.5, and 15 min [57], suggesting that no further biofilm removal occurred after 5 min of exposure. Likewise, it has been reported that the killing of bacteria in the *E. faecalis* biofilms was fastest during the first 3 minutes and slowed down greatly after 10 minutes [20].

Radcliffe et al. [6] studied the minimally required exposure time to eradicate *E. faecalis*, suggesting that with 0.5% NaOCl as irrigant, an exposure time of 30 min reduced viable count to zero, compared with 10 min for 1%, 5 min for 2.5%, and 2 min for 5.25%. Similarly, Tawakoli et al. [58] reported that a multispecies biofilm structure was almost entirely dissolved after 1 min of exposure to 5% NaOCl.

The tissue-dissolving capacity of NaOCl was also reported to be time-dependent [59]. When bovine pulp tissue was immersed in 5% NaOCl for 5, 15, and 30 min, the percentage of tissue dissolution was higher in the 15 min group than that of the 5 min group, while no significant difference was observed between 15 min and 30 min exposure, probably due to the exhaustion of free available chlorine [60]. Stojicic et al. [39] revealed that 2% NaOCl had no obvious tissue dissolution capability in 5 min exposure to organic tissue, as the weight loss of tissue was equivalent to that of distilled water. This is in consistent with another study which reported that the 1.5% NaOCl caused tissue dissolution similar to saline [61]. These results suggested that NaOCl at low concentrations had limited organic tissue dissolution capability with brief exposure, which might be compensated by increasing the concentration or applying agitation techniques [30, 35, 39].

### 4.2. Refreshment

NaOCl demonstrates its antibacterial and tissue-dissolving activity through the interaction between free available chlorine (active chlorine) and the organic composition of bacteria biofilm/residual pulp tissue [62]. However, as the interaction takes place, the amount of free available chlorine molecules decreases, as most of the solvent ability of 2% NaOCl was lost after 2 minutes of exposure to organic materials [34].

Refreshment of NaOCl irrigant is considered a practical method to maintain its efficacy by compensating the loss of free available chlorine during the oxidating process [63]. A numerical model of diffusion efficacy suggested that the diffusion could be enhanced by constantly applying fresh irrigant in the proximity of isthmus or lateral canal-like structure [64]. The feasibility of this method was confirmed by a study of Pereira et al. [57], which demonstrated that one or two refreshments removed significantly more biofilm from the isthmus-like structure, whereas biofilm removal from simulated lateral root canal was unaffected. In addition, some researchers claimed that refreshment could compensate the loss of chemical efficacy of lower concentration of NaOCl, thereby reducing the necessity of the application of NaOCl at high concentrations [23]. Macedo et al. [65] reported that the reaction rate of 2.5% NaOCl with refreshment is approximately five times less than that of 10% NaOCl without refreshment, suggesting that the loss of efficacy at lower concentration could not be compensated by refreshment alone.

### 4.3. Concentration

NaOCl irrigant is used at concentrations varying between 0.5% and 6% with no consensus for the optimal concentration [3]. Dumitriu and Dobre [66] found that concentration of NaOCl was positively correlated to the rate of collagen dissolution. In addition, Stojicic et al. [39] reported that weight loss (dissolution) of the tissue increased almost linearly with the concentration. It has been betoken that high concentrations of NaOCl (5.25%-6%) demonstrated significantly greater antibacterial efficacy compared to mild concentrations (2%-2.5%) [19, 20, 67], which in turn demonstrated significantly greater antibacterial efficacy compared to lower concentrations (1%-1.5%) [68, 69]. Swimberghe et al. [11] reported significantly different biofilm-eradication effectiveness between NaOCl concentrations at 0.025, 0.1, 0.5, and 2.5% for both anaerobically and aerobically incubated *E. faecalis* biofilms. Moreover, some studies suggested that high concentrations of NaOCl are necessary to eliminate bacterial biofilms. Golob et al. [70] proposed that effective and durable decontamination could not be achieved without the application of NaOCl at high concentration (5%), as bacteria managed to regrow 48 hours after treated by 1% and 3% NaOCl. Another study revealed that 1% NaOCl partially reduced *E. faecalis* vitality in the biofilm while the 2.5% and 5.25% NaOCl solutions caused complete inhibition of the biofilm bacterial growth [16].

Penetration depth of NaOCl is vital to the disinfection within dentinal tubules. It was found that the increase of NaOCl concentration resulted in deeper penetration depth, with the observed depth of 1% NaOCl was approximately 60%-80% of that of 6% NaOCl [71]. Likewise, Palazzi et al. [72] reported that the penetration depth was generally increased with increasing NaOCl concentration (1%, 2%, 4%, 5.25%, and 6%), while statistically significant difference was found only between 1% and 6% NaOCl. By contrast, Faria et al. [73] reported that no significant increase of the penetration depth was observed by increasing NaOCl concentration from 2.5% to 6%.

Although the application of concentrated NaOCl presented considerable disinfecting capacity in the *in vitro* studies described above, it has been reported that biofilm removal from isthmus and lateral canal during syringe irrigation was independent from NaOCl concentration [74]. In addition, Verma et al. [75] found that there was no difference in the healing rate or postoperative pain between the high-concentration (5%) and low-concentration (1%) groups, suggesting that the increase of NaOCl concentration did not result in a significance in clinical outcome. Therefore, more studies are required to verify the necessity of the application of high-concentration NaOCl irrigant in clinical practice.

### 4.4. pH

pH value significantly influences the chemical properties of NaOCl irrigant by altering the relative ratio of OCl- and HOCl [76]. The decrease in pH value leads to a higher proportion of HOCl, which is a strong antiseptic, resulting in greater antibacterial efficacy of the irrigant, while its tissue-dissolving capacity would be reduced [38, 43, 77, 78]. The exact opposite effect could be observed when the pH value increases [25, 76]. Dentin has been supposed to influence pH of the NaOCl irrigants via the buffering capacity. Macedo et al. [79] found that the exposure time with dentin and the concentration of the NaOCl solution significantly influence the pH of the solution; however, the observed change in the pH level is too limited to induce a biological effect. Neutralizing or stabilizing the pH value of NaOCl irrigant has been proposed as strategy of achieving ideal chemical properties [25]. Notwithstanding, it is questionable whether the alteration of chemical properties of NaOCl by modifying its pH value is of clinical significance, while the cytotoxicity and impact on mechanical properties of such alteration have not been fully investigated [80].

### 4.5. Gel Form

The application of NaOCl gel, instead of solution, has been proposed with the objective to reduce the risk of apical extrusion and thereby preventing NaOCl accident [81]. Zand et al. [82] reported that the NaOCl gel was as effective as NaOCl solution along with EDTA in terms of smear removal in all parts of root canal walls. Several studies also demonstrated that the NaOCl gel had similar antibacterial efficacy compared to the solution form [67, 83, 84]. Thus, NaOCl gel has potential to be a safe and practical alternative to NaOCl solution during chemical preparation. However, it has been reported that the penetration of NaOCl gel into dentinal tubules was less than the solution form [73]. Moreover, a systematic review concluded that NaOCl gel was less efficient as root canal irrigant, though the evidence of such deduction is considered insufficient [85]. The distribution of NaOCl gel in the complexities of root canal system is of great significance to the clinical outcome of chemical preparation, which is yet to be investigated.

## 5. Methods of Improving the Chemical Effectiveness of NaOCl

### 5.1. Surface Active Agent

One of the drawbacks of NaOCl as root canal irrigant is its high surface tension, which limits its ability to penetrate the root canal irregularities and dentinal tubules [86]. Endeavor to surmount this problem has been made via the introduction of surface active agent in conjunction with NaOCl irrigant.

Surface active agent resulted in lowered contact angle, while the amount of free available chlorine, cytotoxicity, and antibacterial efficacy of NaOCl solution was unaffected [87, 88]. However, experiments investigating the influence of the addition of surfactants on the penetration depth of NaOCl irrigant, the fundamental purpose of the application of surface active agent, have shown controversial results. A recent study showed higher penetration depth into dentinal tubules when surfactant was added into 3% NaOCl [89], while two studies found the opposite as the addition of surfactant did not increase the penetration depth of 2.5% or 5.25% NaOCl solutions into dentinal tubules [73, 90]. In addition to the penetration into dentinal tubules, the application of surfactant was reportedly to enhance the tissue dissolution capability of NaOCl as a result of the low pH, since organic dissolution is greater at an alkaline pH [91]. Root canal irrigation with 5% NaOCl has been proved to cause significant decrease of dentin microhardness, while the loss could be restored after the following irrigation with 1% benzalkonium chloride supplemented EDTA. Benzalkonium chloride is a surfactant that enhances the antimicrobial action of EDTA and its wettability on dentin [92]. Generally, the application of surfactant has been associated with greater tissue dissolution by NaOCl [93–95], whereas the removal of hard tissue debris remained altered [96].

### 5.2. Continuous Chelation

Continuous chelation is an innovative root canal irrigation protocol where NaOCl is mixed with an etidronate powder (HEDP) to create a new endodontic irrigant [97]. HEDP is a weak chelator and compatible to NaOCl solution [98]; this combination could serve as an alternative to the current NaOCl/EDTA protocol since the new solution was reported to reduce dentin erosion and demonstrated homogenous distribution of organic and inorganic contents on the root canal surface after irrigation [99, 100]. However, current studies demonstrated conflicting results concerning the influence of the combination of HEDP on the chemical properties of NaOCl and the mechanical properties of treated dentin. Ulusoy et al. [101] revealed that the application of HEDP was associated with higher fracture resistance compared to EDTA, whereas statistically lowered fracture resistance and nanohardness were reported when HEDP was applied [100, 102]. In terms of root canal cleanliness, HEDP was found to be effective in dissolving and killing bacteria in conjunction with NaOCl [103]; the antibacterial effectiveness of NaOCl+ HEDP was even reported to be greater than that of NaOCl+ EDTA [104]. However, no difference in removal of smear layer and dentin debris was found between these two irrigation strategies [105]. Though this innovative irrigation protocol has been proved to be practical and safe, its clinical efficacy remained a matter of debate [106, 107].

### 5.3. Heating

Heating NaOCl irrigant could enhance the chemical properties of NaOCl by increasing its reaction rate [66]. Warming of NaOCl could be done either by heating the irrigant inside root canal or preheating before use, with the former approach being more effective [108].

It was reported that greater tissue dissolution, bacteria elimination, and smear layer removal could be achieved by heating NaOCl while maintaining its free available chlorine [39, 109]. Iandolo et al. [110] found that intracanal heating significantly enhanced NaOCl penetration into dentinal tubules. Furthermore, a study conducted by Yared and Al Asmar Ramli [111] suggested that the improvement of antibacterial ability caused by heating NaOCl was greater than that of the application of agitation techniques including sonic and ultrasonic irrigation. However, some studies demonstrated that the heating of NaOCl did not result in an increase of reaction rate and tissue-dissolving capability of NaOCl irrigant [65, 112], nor did the temperature influence the penetration depth of irrigant into dentinal tubules [71, 72]. Xu et al. [69] reported that the temperature of NaOCl irrigant did not significantly influence the biofilm eradication efficacy during mechanochemical irrigation conducted in a biofilm reactor. These conflicting results might be attributed to variation in the tested temperature and concentration of the heated irrigant. In addition, since an increase of temperature of NaOCl irrigant could be achieved by applying agitation technique such as laser or ultrasonic devices [113], the necessity and effectiveness of heating NaOCl under different scenarios are yet to be explored.

### 5.4. Octenidine Dihydrochloride (OCT)

Due to the presence of organic content such as dentin collagen and microbial biomass, the efficacy of NaOCl irrigant would be compromised, resulting in bacterial persistence [3]. While this could be addressed by increasing the concentration of NaOCl, the increase in cytotoxicity and decrease in mechanical properties of treated dentin are additional concerns [114, 115]. OCT is an antiseptic with a broad spectrum of antibacterial properties [116]; the combination of OCT and NaOCl might be a strategy to enhance biofilm eradication [3]. The interaction between OCT and NaOCl leads to the formation of phenoxyethanol [117], an antimicrobial agent, without affecting the free available chlorine of NaOCl [118]. The conjunction of OCT reduced the cytotoxicity of 5.25% NaOCl [119], demonstrating potential for combined irrigation.

## 6. Agitation Techniques That Enhance the Efficacy NaOCl

Traditionally, NaOCl is delivered into root canal system by syringe, referred to as traditional needle irrigation (CNI), which failed to demonstrate satisfactory irrigating effectiveness [3]. Therefore, several agitation techniques have been developed to achieve sufficient disinfection and debridement of the root canal system.

Passive ultrasonic irrigation (PUI) is a type of ultrasonic-driven irrigation that has become one of the most widely used technique to activate NaOCl irrigant in clinical practice [120]. Passive sonic irrigation (PSI) devices, including EndoActivator (EA) and EDDY, are equipped with noncutting plastic tips that could reduce the risk of changing root canal morphology by inevitably contacting the root canal walls [121]. Laser has also been proposed to activate NaOCl irrigant during chemical preparation, known as laser-activated irrigation (LAI) [122], where Er:YAG laser demonstrated the most promising efficacy. When applied with subablative energy (20 mJ and 15 Hz) and ultrashort pulses (50 *μ*s), Er:YAG laser could induce intracanal cavitation and shockwaves as a result of photoacoustic and photomechanical effects, known as photon-induced photoacoustic streaming (PIPS) [123]. Recently, a shock wave-enhanced emission photoacoustic streaming (SWEEPS) technique has been developed to further improve the irrigation efficacy of LAI [124]. XP-endo Finisher (XPF) is a size 25 nontapered rotary instrument produced using nickel-titanium (NiTi) MaxWire alloy. The file has a straight shape (M phase) at room temperature whereas it develops a phase change into spoon-like shape (A phase) when exposed to body temperature; this unique shape increases the chances to touch canal walls and agitate irrigant when rotating in the canals. The GentleWave (GW) System is a novel irrigation device developed to clean and disinfect root canal system with minimized root canal enlargement, since the tip is positioned only in the pulp chamber [125]. Negative pressure irrigant delivery devices have been introduced to endodontic therapy to avoid NaOCl accident, an example of which is EndoVac, which is capable of creating constant irrigant flow up to the working length and contributing to debridement and disinfection in the apical region [126].

In this segment, we compared the differences of the aforementioned agitation techniques in terms of their abilities to enhance the efficacy of NaOCl, including the tissue-dissolving and antibacterial properties, the penetration and distribution in the root canal system, the removal of dentin debris and smear layer, and the clinical outcomes.

### 6.1. Tissue Dissolution

The removal of pulpal remnant and organic tissue is of great significance to the success of root canal treatment, the achievement of which depends on the application of NaOCl [24], which is the only clinically acceptable irrigant that could dissolve organic tissue [127]. Agitation is a detrimental factor that could enhance the tissue-dissolving capacity of NaOCl irrigant [39].

GW has been proven to be a very effective agitation technique in terms of tissue dissolution. Compared to PUI and EA, GW demonstrated a tissue dissolution rate that was more than 8 times faster than the second fasted device using 6% NaOCl as irrigant [127]. In addition, NaOCl irrigant activated by Er:YAG laser was also reported to be highly efficient in soft tissue dissolution [128]. Guneser et al. [24] compared the pulp tissue-dissolving ability of Er:YAG laser with endodontic fiber tip, PIPS, and EA; the results showed that both of the LAI techniques revealed significantly higher tissue dissolution rate than EA and nonactivated group, whereas Er:YAG laser with endodontic fiber tip was found to be more effective than PIPS. Interestingly, tissue dissolution rate of 5.25% irrigant was not improved by EA compared to the nonactivated group; this was in accordance with a previous study which revealed that EA had no effect on tissue-dissolving capacity of the activated NaOCl irrigant [41]. These unsatisfactory results might be attributed to the low energy of sonic devices and insufficient irrigant streaming [24].

The removal or dissolution of inflamed pulp tissue is essential in the treatment of internal root resorption cases [129]. PUI demonstrated similar efficacy with sonic devices, while being more effective than CNI in terms of tissue dissolution in simulated grooves in root canals [95, 130]. Furthermore, XPF was found to be more effective compared to PUI regarding the removal of organic tissue in artificial internal resorption cavities [42].

### 6.2. Antibacterial Effectiveness

PUI is an effective irrigation technique that improves the action of NaOCl against root canal bacteria and the endotoxins [131–133]. However, it remained controversial whether PUI could exhibit greater antibacterial efficacy than CNI [134, 135]. A recent systematic review concluded that only around 50% of the *in vitro* studies found a superior antimicrobial effect compared to syringe irrigation [136], while another systematic review reported poor evidence regarding the relatively stronger disinfecting capacity of ultrasonic-activated NaOCl irrigant compared to the nonactivated counterpart [137].

Compared to PUI, PSI demonstrated similar antibacterial efficacy in both curved and straight root canals [138]. Moreover, the two sonic-powered irrigation devices, EA and EDDY, have presented similar effect, both of which were more effective than the nonactivated group [139]. Zeng et al. [140] revealed that although EDDY had better intratubular bacterial killing efficacy than CNI in the coronal and middle part of the root canal, no difference was found in the apical region. In addition, EDDY did not provide additional effect over CNI in biofilm eradication from deep intraradicular dentin using 3% NaOCl as irrigant, suggesting that the difference in dentin disinfection between EDDY and CNI could only be discerned up to the penetration depth of 100 *μ*m.

Neelakantan et al. [141] compared the effectiveness of LAI and PUI during chemical preparation and reported that LAI was superior to PUI in terms of dentinal tubule disinfection. Moreover, PIPS resulted in greater effectiveness in dentinal tubule disinfection compared to sonic irrigation and conventional needled irrigation [142]. However, Race et al. [143] reported that no significant difference was observed between LAI and PUI on eradicating a mixed-species biofilm in human mesial roots. The result might be attributed to the low power settings (0.5 W and 0.75 W) and high NaOCl concentration (4%); both parameters could significantly influence the fluid dynamics during LAI and might impede the irrigant into the root canal complexities [144, 145].

XPF has also demonstrated promising antibacterial effectiveness compared to PUI and CNI [146]. Azim et al. [142] reported that XPF was more effective than PIPS, EA, and PUI in disinfecting the main canal space. Alves et al. [147] found that when XPF and PUI were applied to activate 2.5% NaOCl, only the former could significantly reduce the bacterial count. A more recent study revealed that PUI resulted in significantly lower biofilm volumes than XPF did in the coronal and middle parts of the root canals, whereas no difference was found between these two techniques in the apical third [148]. With regard to isthmus disinfection, PUI presented statistically lower biofilm volumes in the coronal third, with no difference between the two activation systems in the middle and apical parts [148].

The EndoVac apical negative pressure irrigant delivery system was found to be effective compared to CNI in terms of antimicrobial efficacy [149–151]. It was reported that EndoVac was even more effective than PUI regarding the elimination of E. faecalis in root canals [152]. More studies are required to compare the antibacterial efficacy of EndoVac with other agitation techniques, such as LAI and PSI.

As a novel activation device, GW demonstrated promising antibacterial effectiveness. Zhang et al. [125] revealed that GW resulted in higher reduction in bacterial DNA compared to PUI using 3% NaOCl as irrigant. Furthermore, greater biofilm removal from root canals of mandibular and maxillary molars was observed in the GW-treated group than those treated with PUI protocol [153].

### 6.3. Penetration

It has been clearly demonstrated by the microbiology of endodontic infection that bacteria can be found in main canal space, lateral canals, and dentinal tubules [71]. The penetration and distribution of NaOCl irrigant are crucial to the eradication of bacteria in these areas [3].

Several agitation techniques have been verified to significantly increase NaOCl penetration into dentinal tubules, including PUI, PSI, LAI, and EndoVac [73, 110, 126, 154, 155]. Akcay et al. [156] investigated the effect of different agitation techniques on the dentinal tubule penetration of 5% NaOCl solution; the result demonstrated that PIPS was the most effective activation system followed by PUI, EA, and CNI, with statistical difference among the groups. In addition, it was reported that PIPS resulted in significantly greater penetration depth of irrigant into the dentinal tubules in the apical region compared to PUI, EDDY, CNI, and nonactivated control group [157, 158]. This could be attributed to the capability of PIPS to create considerable fluid dynamics away from the laser tip [159]. However, SWEEPS did not show additional effect on dentinal penetration of irrigant in the apical third compared to CNI, potentially compromising the dentin disinfection in this region [157, 158].

In addition to the penetration into the dentinal tubules, the distribution of NaOCl irrigant in the root canal system is also essential to the success of chemical preparation. It was reported that XPF was more effective in distributing irrigant throughout the mesial root canal system compared to PUI and CNI [160]. Furthermore, Merino et al. [161] reported that PUI was more effective in the delivery of NaOCl up to the working length compared to EA. Among the four tested irrigation systems including EA, PUI, EndoVac, and F file, EndoVac was found to be the most effective in reaching the working length, whereas PUI was superior at lateral canal penetration [162]. This was in accordance with a previous study which demonstrated that PUI and EA resulted in significantly more penetration of 5.25% NaOCl irrigant into the lateral canals in comparison with CNI [163].

### 6.4. Canal Cleanliness

The main purpose of root canal preparation is the cleaning and shaping of root canal system, the cleanliness of which is dependent on the proper removal of hard tissue debris and smear layer [164]. Several agitation techniques, including EndoVac, PUI, PSI, and GW, have been reported to be effective in smear layer removal and debridement [164–169]. However, studies regarding the efficacy of LAI in canal cleansing have demonstrated conflicting results. Several studies showed that LAI resulted in significantly higher reduction in smear layer and debris after irrigation compared to CNI [170–175], even in the apical region [176], whereas other studies found the opposite as LAI presented no additional effect [177–179]. Yang et al. [180] revealed that SWEEPS irrigation resulted in significantly less hard tissue debris in the root canal system compared to PIPS and PUI, with no difference between these two groups. Similar results were also reported as debridement caused by SWEEPS was comparable to conventional laser-activated, whereas both of which were more effective than PIPS and PUI [181].

### 6.5. Clinical Outcome

Although a great number of *in vitro* studies have evaluated the effectiveness of activation methods, *in vivo* studies are required to provide higher-level evidence regarding the performance and benefits of irrigation devices. Liang et al. [182] compared the radiographic healing after root canal treatment with and without additional ultrasonic activation of 5.25% NaOCl, with no significant difference was found between CNI and PUI. A serial experiment revealed that the 6-month and 12-month healing rates after GW irrigation using 3% NaOCl irrigant were 97.4% and 97.3%, respectively [183, 184]. Moreover, for those cases with sizable periapical lesions, treatment with GW had a high healing rate of 97.7% at 1-year reevaluation [185]. More randomized controlled trial and prospective studies are warranted to investigate the clinical efficacy of the activation of NaOCl irrigant by different agitation techniques.

## 7. Side Effect

### 7.1. Apical Extrusion

Apical extrusion, known as hypochlorite accident, is defined as the incidence which NaOCl irrigant extrudes beyond the apex, with or without dentin debris [186]. Large apical foramina, root resorption, and misplacement of the needle could lead to this complication [187]. Since NaOCl is cytotoxic to periapical tissues, the extrusion of NaOCl may cause an inflammatory reaction [23], which leads to tissue damage and pronounced symptomatology [188]. Typical symptoms of apical extrusion include persistent pain, swelling, or even buccal bone plate defect [189, 190]. Azim et al. [191] compared NaOCl extrusion caused by five irrigation systems; the results demonstrated that apical extrusion seemed unavoidable except for negative pressure irrigation systems such as EndoVac. Interestingly, PIPS resulted in significantly more apical extrusion compared to other irrigation systems [191]. Another study also demonstrated that LAI resulted in significantly higher apical extrusion compared to PUI, with no difference between PIPS and SWEEPS [181]. These results were at variance with other laboratory studies that showed comparable or less irrigant extrusion between LAI and other agitation techniques [145, 192–194]. Meanwhile, studies regarding apical extrusion caused by PUI have demonstrated similar results, suggesting that PUI leads to less or similar irrigant extrusion compared to syringe irrigation [195–197]. It is noteworthy that the relatively less amount of apical extrusion of EndoVac has been widely reported [126, 191, 195], while the application of NaOCl gel, instead of solution, is regarded as another strategy to reduce the risk of apical extrusion [81, 198]. Since apical extrusion of NaOCl irrigant could not be measured *in vivo*, the data obtained *in vitro* studies cannot be directly extrapolated to conditions in clinical practice.

### 7.2. Cytotoxicity

As a strong oxidizing agent, NaOCl is cytotoxic to the periapical tissue and stem cells, especially in case of regenerative endodontic treatment [199]. The cytotoxic effect to dental stem cells was reported to be concentration dependent [115, 200]. Furthermore, Martin et al. [201] found that dentin conditioning with 1.5% NaOCl resulted in greater survival and differentiation of stem cells of apical papilla compared to the 3% counterpart, whereas 6% NaOCl leads to lack of survival and differentiation. Therefore, the application of lower concentrations of NaOCl was advocated in regenerative procedures.

### 7.3. Postoperative Pain

A major complication of root canal treatment is the occurrence of postoperative pain caused by various factors, one of which is the extrusion of NaOCl irrigant [202]. Due to its cytotoxicity, the extruded NaOCl could trigger an acute inflammatory reaction in the periapical tissue, resulting in postoperative pain that mostly occurs 24 h after treatment [203]. The concentration of NaOCl irrigant is a major factor in terms of the prevalence of postoperative pain, as the intensity and frequency of postoperative pain were reported to significantly decline by reducing the NaOCl concentration from 5.25% to 1.3% [204]. However, Farzaneh et al. [205] found the opposite where 5.25% NaOCl was associated with significantly lower postoperative pain compared to 2.5% NaOCl. The conflicting results might be ascribed to the difference inclusion criteria, with the latter experiment specifically included patients without periapical pathosis, potentially reducing the risk of irrigant extrusion. Ulin et al. [206] reported that the concentration of NaOCl (0.5% vs. 3%) did not affect neither the frequency nor the magnitude of postoperative pain, while significantly higher incidence of postoperative swelling was recorded when a greater concentration of NaOCl was applied.

Theoretically, as mentioned above, the application of NaOCl gel is an alternative that could reduce the risk of apical extrusion and thereby reducing the occurrence of postoperative pain. A study regarding this topic showed that the NaOCl gel group resulted in significantly less postoperative pain compared to its solution counterpart within the first 24 h, while no significance difference was recorded on days 2, 3, and 7 [207]. More randomized controlled trials are required to verify the potential of NaOCl gel to reduce postoperative pain.

Surprisingly, although it was claimed that the activation of irrigant might lead to a greater amount of apical extrusion [191], current studies suggested that the application of agitation techniques, including LAI, PUI, GentleWave, and EndoVac, results in less or similar postoperative pain compared to syringe irrigation [208–210], with no significant difference among these activation methods [203, 210, 211]. This provides evidence for the equivocal results regarding the apical extrusion caused by different agitation techniques.

### 7.4. Adverse Effect on Mechanical Properties of Dentin

The irrigation protocol using NaOCl irrigant would impose a time- and concentration-dependent adverse effect on mechanical properties of dentin [212], such as microhardness and elastic modulus, which is mainly attributed to NaOCl instead of EDTA [213]. The application of NaOCl leads to the formation of an appetite-rich, collagen-sparse dentin subsurface, referred to as “ghost layer” [214]. Increase of exposure time (11.5 min vs. 19 min) and concentration (1.3% vs. 5.25%) resulted in decrease in fracture resistance and elastic modulus, respectively [215, 216]. It has been reported that the application of 2.5% NaOCl for 60 s did not alter the dentin microhardness [217], while 0.5% NaOCl gel caused a significant decrease in flexural strength and microhardness in 7 days [114]. Moreover, the dentin microhardness was reduced after exposure to 5.25% NaOCl for 5 min, whereas the flexural strength was not affected [218]. The deleterious effect of NaOCl irrigant seems to differ depending on the outcome evaluated which may be responsible for the variation in experimental results. Interestingly, ultrastructural alteration caused by NaOCl was found to be aggravated by PUI [219], whereas another study found that the microhardness of dentin treated with NaOCl was not affected by PIPS [217]. The influence of activation techniques on mechanical properties of treated dentin warrants further studies.

### 7.5. Generation of Harmful Volatile Compounds during Chemical Preparation

Due to the reaction of chlorine or hypochlorite with natural organic matter (NOM) such as pulpal tissue and bacterial biofilm, harmful volatile organic compounds (VOCs) and chlorinated disinfection by-products (DBPs) can be generated during chemical preparation with NaOCl. The carcinogenic and mutagenic properties of these VOC and DBP compounds may further impair human health via leaking through the apical foramen, biologically interacting with periapical tissue and even entering blood circulation [220]. By using an *ex vivo* experimental model, Ioannidis et al. [221] found chemomechanical preparation of artificially infected root canals with Ni-Ti rotary instruments, along with 2.5% NaOCl in combination with 17% EDTA caused the release of high concentrations of methanol, propanol, ammonia, chloroform, and formaldehyde. The related potential environmental and health hazards of the significant generation of these VOC and DBP compounds should be further investigated.

## 8. Conclusion

NaOCl plays an indispensable role in chemical preparation of root canal treatment. *In vitro* studies suggested that the characteristics of bacterial biofilms could affect their susceptibility against NaOCl. In addition, the efficacy of NaOCl could be affected by various parameters such as concentration, temperature, exposure time, and pH value of the irrigant. Numerous techniques have been developed to activate NaOCl since the application of NaOCl alone could be ineffective during chemical preparation, though controversy remained regarding their clinical effectiveness. Despite its antibacterial and tissue-dissolving capacities, NaOCl possesses cytotoxicity against periapical tissue whereas the strong oxidant property would impose negative impact on the mechanical properties of dentin. Therefore, NaOCl should be used with caution to avoid its detrimental effect in clinical practice.

We recommend that future studies should focus on the disinfection in apical third and deeper layer of dentin, since these areas are potential harbors of bacterial biofilms. Furthermore, more clinical studies are warranted to provide due credence to the efficacy of agitation techniques.

## Data Availability

Data sharing is not applicable to this article as no new data were created or analyzed in this study.
